# Supplementary material for: Imp is required for timely exit from quiescence in Drosophila type II neuroblasts
Source: PLoS One. 2022 Dec 15;17(12):e0272177. doi: 10.1371/journal.pone.0272177 (PMC9754222; doi:10.1371/journal.pone.0272177)
Supplement: Attachment — Submitted filename: _reviewer response.docx [file pone.0272177.s001.docx]

Reviewer #1: Balancing the proliferation and quiescence is essential for the normal functions of stem cells. In this manuscript, Munroe et al investigated the role of Insulin-like growth factor II mRNA- binding protein (Imp) in proliferation and quiescence of Drosophila type II neuroblasts (TIINBs). They found Imp displayed a high-to-low temporal gradient in TIINBs and the Imp gradient can be effectively shifted to different times in development by ImpRNAi and Imp overexpression. They further suggested that pnt-Gal4 UAS-GFP could be used as a marker to identify proliferating type II neuroblasts. By quantifying the number of proliferating TIINBs, they found that Imp knockdown delayed exit from quiescence, whereas overexpression of Imp did not induce precocious exit from quiescence in TIINBs. Moreover, comparable levels of Imp were observed in quiescent and proliferating TIINBs while its antagonist protein Syp expressed at a significantly lower level. Overall, this paper suggests a necessary but not sufficient role of Imp in mediating timely exit from quiescence in TIINBs. Experiments were carefully performed, the data presented are of high quality, and interpretations of the results are comparatively justified. I only have several minor suggestions for the authors to consider:

1. For all the Imp/syp immunostaining data, the numbers of TIINBs for statistical analysis were not provided in Methods or Figure legends.

Thank you for the observation, in response we have added quantification of brain number and neuroblast number to the relevant figure legends.

2. Line 133,“Syp levels in quiescent TIINBs were slightly higher than Syp levels in proliferative TIINBs (Figure 4F), showing a correlation between higher Syp levels and neuroblast quiescence.” Whereas the figure legend of Fig4E claimed that “Imp and Syp levels are the same in quiescent and proliferating type II neuroblasts. “Based on the data, the legend needs to be fixed.

We agree, and have made the following change in the figure legend of Fig4E: "... Syp levels are lower in proliferating type II neuroblasts."

Reviewer #2: Munroe et al. report a role for Imp in exit from quiescence of type II neuroblasts in Drosophila. They examine and quantify Imp protein levels in Type II neuroblasts at different larval stages, something that had been missing (although much needed) in the field. They also test whether Imp regulates exit from quiescence based on high to low Imp expression and based on the known role of Imp in regulating neuroblast" decommissioning". While the data presented in this manuscript is solid and well presented, this reviewer has one point that may need to be further addressed:

Authors report that Pnt-Gal4 is only active in proliferating type II neuroblasts. By this logic, using Pnt-Gal4 should only knockdown Imp in proliferating neuroblasts. Is knockdown of Imp delaying the exit from quiescence or suppressing proliferation? or both? Is there data to suggest that Pnt-GAL4/UAS transgene expression perdures in quiescent type II neuroblasts?

Great point! We've added the following text to the Discussion. "It is interesting that Pnt-gal4 is not expressed in quiescent neuroblasts, yet is able to drive UAS-ImpRNAi at sufficient levels to maintain quiescence. Type II NBs are proliferative in the embryo, then go quiescent, and normally resume proliferation in 12-30h old larvae. We propose that Pnt-ga4 is expressed in the embryo type II neuroblasts where it drives UAS-ImpRNAi which persists into larval stages due to perdurance of Gal4 and ImpRNAi, thus extending quiescence. As ImpRNAi levels begin to rise (due to lack of Pnt-Gal4 UAS-ImpRNAi expression) the neuroblasts resume proliferation. We see no evidence for a second wave of quiescence due to re-expression of Pnt-gal4."

Typo: Line 66, It is the non-mushroom body neuroblasts that require low Notch signaling to be driven out of quiescence. MB neuroblasts do not enter and exit quiescence. Authors should also check the reference for this statement.

Thanks for catching this error; we have changed the text to say "previous work has shown that Syp recruits the mediator complex and Pros to drive the MB NBs into decommissioning (21)."

Reviewer #3:

Overall evaluation and significance

Munroe et al., investigate the role of Imp, an important RNA-binding protein (RBP) involved in Drosophila neurogenesis, in neuroblast (NB) quiescence exit. Re-entry of NBs into cell cycle has been previously shown to be regulated by nutrition-dependent glial niche and the Notch signaling pathway, while the role of Imp in this process has not been explored. Here, the authors characterised the kinetics of Type II NB quiescence exit upon modulation of the Imp expression gradient. They observed that reducing the level of Imp, which accelerates Imp protein depletion in Type II NBs, delays exit from quiescence, while the overexpression of Imp did not lead to a noticeable effect. Taken together, the authors suggest a previously unknown role of Imp in promoting re-entry of quiescent NBs into cell cycle. The manuscript is high quality and of value and interest. It should be accepted for publication, once the authors have addressed our concerns, either by revising the text or if they would prefer, providing additional data.

Major comments

The authors first present a detailed comparison of Imp expression kinetics in Imp RNAi and Imp OE experiments. The study then aims to demonstrate that modulating Imp affects Type II NB quiescence exit. However, their choice of PntP1-GAL4 driver needs to be explained / justified, in light of the main conclusion of this work.

1. Throughout the manuscript, the authors utilise PntP1-GAL4 line to identify proliferating NBs and to drive UAS transgenes. However, in Figure 3, the authors describe that PntP1-GAL4 only becomes active in proliferating NBs and not in quiescent cells. Therefore, the Imp RNAi should be inactive in quiescent NBs, which does not support the authors' conclusion that reduced level of Imp leads to delayed quiescence exit. The authors should include more information that justifies their choice of PntP1-GAL4 over more conventionally used wor-GAL4, ase-GAL80.

Great point! We've added the following text to the Discussion. "It is interesting that Pnt-gal4 is not expressed in quiescent neuroblasts, yet is able to drive UAS-ImpRNAi to maintain quiescence. Type II NBs are proliferative in the embryo, then go quiescent, and normally resume proliferation in 12-30h old larvae. We propose that Pnt-ga4 is expressed in the embryo type II neuroblasts where it drives UAS-ImpRNAi which persists into larval stages due to perdurance of ImpRNAi, thus extending quiescence. As ImpRNAi levels begin to rise (due to lack of Pnt-Gal4 UAS-ImpRNAi expression) the neuroblasts resume proliferation. We see no evidence for a second wave of quiescence due to re-expression of Pnt-gal4."

2. Although the knock down of Imp in Figure 2C-D seems convincing on the population-wide scale, a key piece of data is missing that GFP-negative quiescent NBs are under PntP1-GAL4/Imp-RNAi control (Figure 4A). Can the authors provide an explanation or data that Imp levels are reduced in quiescent NBs in Imp RNAi conditions compared to the wild-type at 24-48 ALH, and also discuss why Imp RNAi is active despite the lack of GFP expression? Is it possible that other cell types (e.g. glia) might be affected by the driver?

Thank you for the observation and we can see that this point was not made clear in the text. We have added the following text to the methods: "We were unable to quantify Imp fluorescence in quiescent TIINBs in ImpRNAi flies because quiescent TIINBs cannot be distinguished from quiescent Type I neuroblasts."

3. Are all PntP1>GFP-positive NBs CycE-positive? The population of GFP-positive but CycE-negative cells may suggest that quiescence exit is a multi-step process where Imp plays a role in the initial step.

We are sorry that this was not made clear, the following change has been made to the text on page 4. "…proliferative neuroblasts in interphase are GFP+Dpn+CycE+ whereas quiescent neuroblasts are GFP-Dpn+CycE- (15,16)."

Minor comments

1. Figure 2A-B: Representative wild-type series of Type II NB images should be provided to match the quantification shown in Figure 2C.

We show the requested wild type stains in Figure 1B-C.

2. Line 213: Please provide replicate information in all figure legends. In particular, what does the symbol represent in Figure 4A and 4C? It should be clear how many brains were quantified per biological replicate.

We changed the symbol “#” to “Number” – i.e. the Y axis shows the number of proliferating type II neuroblasts (of which there is a maximum number of 16 per brain). Replicate information has been added to all figure legends.

3. Figure 4E-F: Syed et al., 2017 (doi.org/10.7554/eLife.26287) have shown lack of Syp expression in Type II NBs at stages before 48 ALH and upon disruption of ecysone signaling. Can the authors explain the discrepancy?

Thank you for this observation. We have made the following change to the results section: “As expected, we found Syp to be expressed at lower levels than Imp in both proliferating and quiescent TIINBs (Figure 4E, third and fourth columns; quantified in 4F). Previous work has shown little to no Syp expression in early TIINBs; the very low levels of Syp seen here may be due to more sensitive acquisition methods than used previously (Syed et al., 2017)”.

4. Line 197: Immunofluorescence quantification method should be explained in more detail. Was the Raw Integrated Density calculated over 3D volume of the NBs or on select 2D planes? Why were nuclear areas and intensities removed from the analysis? Were any background subtraction method used?

We agree, and have made the following changes to the methods: "In FIJI, TIINBs were manually selected in a 2D plane at the largest cross section of the TIINB with the polygon lasso tool, and the area and Raw Integrated Density (RID) was measured. Imp is cytoplasmic and measuring fluorescence in the nucleus functioned as background subtraction."

5. In Figure 2C, how were the fluorescence intensity signals normalised between biological replicates and different genotypes?

Thank you for this comment. We have made this addition in the methods: "Imp is cytoplasmic and measuring fluorescence in the nucleus functioned as background subtraction."

6. Line 168: The role of Syp in entering the embryo-to-larval neuroblast quiescence is not yet established. The text should be revised. Perhaps the authors meant 'decomissioning and cell cycle exit'?

Thanks for catching this error. We have changed the relevant sentence from the discussion. "Interestingly, Syp levels in quiescent TIINBs were higher than Syp levels in proliferative TIINBs, showing a correlation between high Syp levels and neuroblast quiescence, and consistent with earlier work showing Syp is required to elevate levels of nuclear Prospero and initiate decommissioning (21)."
